# Coccidioidal Pulmonary Cavitation: A New Age

**DOI:** 10.3390/jof9050561

**Published:** 2023-05-12

**Authors:** Lovedip Kooner, Augustine Munoz, Austin Garcia, Akriti Kaur, Rupam Sharma, Virginia Bustamante, Vishal Narang, George R. Thompson, Rasha Kuran, Amir Berjis, Royce H. Johnson, Arash Heidari

**Affiliations:** 1 Department of Medicine, Division of Infectious Diseases, Kern Medical Center, Bakersfield, CA 93306, USA; 2 Valley Fever Institute, Kern Medical Center, Bakersfield, CA 93306, USA; 3 Department of Medicine, David Geffen School of Medicine, University of California Los Angeles, Los Angeles, CA 90095, USA; 4 Department of Internal Medicine, Division of Infectious Diseases, UC-Davis Medical Center, Sacramento, CA 95817, USA; 5 Department of Surgery, Kern Medical Center, Bakersfield, CA 93306, USA

**Keywords:** coccidioidomycosis, valley fever, cavity, cavitation, cavitary, coccidioidal

## Abstract

*Coccidioides* species are thermally dimorphic fungi found in geographically defined areas of the Western Hemisphere. The primary portal of entry is respiratory, with symptomatic pneumonic diseases as the most common presentation. Subsequent pulmonary complications as well as extrapulmonary metastatic infection may occur, either of which may be the presenting disease manifestation. Cavitary lung disease may be found incidentally or when investigating symptoms such as cough or hemoptysis. This study aims to explore the spectrum of coccidioidal cavities and the evaluation and management in a cohort of patients seen at Kern Medical over the last 12 years.

## 1. Introduction

*Coccidioides* species are fungal pathogens unique to the Western Hemisphere in the desert southwest of the United States, adjacent to Mexico, and in relatively small foci of Central and South America, although the disease appears to be expanding from traditional regions of endemicity [1,2]. Wernicke and Posadas first described the disease in Argentina in 1892 [3,4]. For many years, only disseminated cases were recognized and described as “coccidioidal granulomas”. The work of Dixon and Gifford in 1935 elucidated that a pneumonic disease of unknown cause termed “San Joaquin Valley Fever” was, in fact, the primary coccidioidal infection and the portal of entry of almost all coccidioidal disease [5]. The identification of coccidioidal cavitation was accomplished shortly after by Farnes and Mills in 1938 [6].

The similarities between pulmonary cavitary coccidioidomycosis and tuberculosis historically have delayed discovery and accurate diagnoses. William Winn, at the Tuberculosis Sanitorium in Springville, California, noted that cases of chronic cavitary coccidioidomycosis were in fact often misdiagnosed as pulmonary cavitary tuberculosis. Much of the putative therapy used for tuberculosis was emulated to treat cavitary coccidioidomycosis and its complications. These therapies included collapse techniques such as pneumothorax and pneumoperitoneum and, later, surgical resection [7]. Winn reported fourteen of ninety-two patients (15.2%) underwent surgical resection. Winn also reported that surgery was commonly utilized for those with large or persistent cavitations, mycetoma, significant hemorrhage, or rupture into the pleural space with empyema [8].

In 1958, Hyde described 211 patients with coccidioidal pulmonary cavitation, of which 108 (51%) underwent surgery. One-third of the surgical interventions in Hyde’s study were performed to ascertain the diagnosis, one-third to control bleeding, and one-third to remove asymptomatic coccidioidal cavities found on chest imaging due to concern for possible malignancy [9].

Significant bleeding may cause several complications, including asphyxia, and management is primarily via: (1) bronchoscopic inspection performed once the airway has been secured and an occlusive catheter such as a Fogarty, placed within the offending airway; (2) interventional radiology embolization/occlusion of the offending vessel(s) feeding the cavitary lesion, usually a branch of a bronchial artery; and (3) as a last resort surgical resection.

In cases of cavitary rupture into the pleural space, treatment always necessitates intervention. Guidelines for coccidioidal cavities from the 2016 Infectious Disease Society of America recommend “prompt decortication and resection of the cavity, if possible.” [10] When there is a delay in diagnosis and treatment, Video Assisted Thoracic Surgery (VATS) or formal thoracotomy may be required for surgical decortication with or without anatomic or non-anatomic lung resection [10].

With the advent of amphotericin B, a further role of medical versus surgical therapy arose with various approaches [11]. Although controversial, some of these included the use of amphotericin B in the perioperative period as summarized by Melick and Grant [12]. The effectiveness of Amphotericin B was less certain than in diseases caused by other fungi [13]. The advent of triazoles has relegated amphotericins to rescue therapy, mainly used in widely disseminated cases, triazole intolerance, or when there are contraindications to triazoles [14].

The purpose of this observational study is to review the demographics, clinical manifestations, and outcomes of patients with coccidiodal pulmonary cavitations in a more diverse population than previously studied [15,16]. Particularly the importance of medical therapy as opposed to the historic use of surgery was evaluated. This study was undertaken at a publicly funded academic institution in the San Joaquin Valley of California.

## 2. Methods

**Figure 1:**
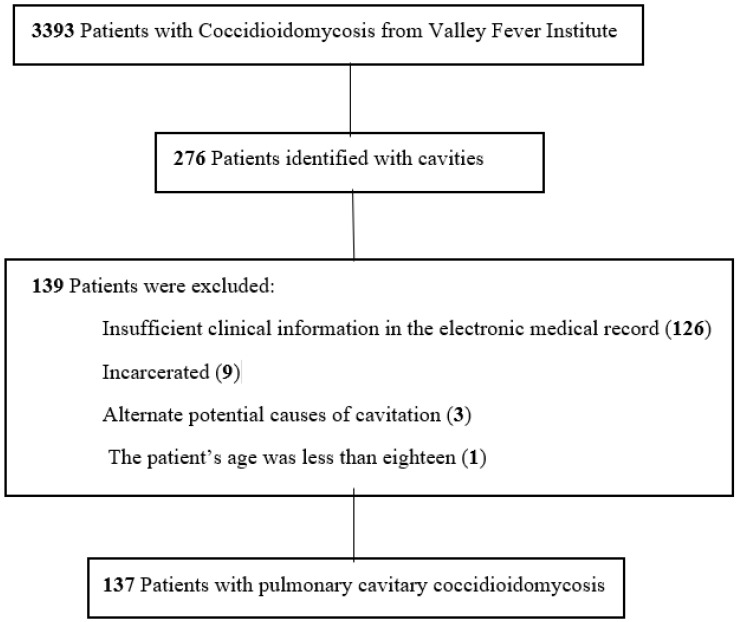
Flowchart of patients with inclusion and exclusion criteria in the study of pulmonary cavitary coccidioidomycosis.

This study was approved by the Kern Medical Institutional Review Board. A waiver of consent was granted, given the deidentified retrospective nature of the project. The Valley Fever Institute database was utilized to identify patients and their electronic medical records at Kern Medical. Records were reviewed for the period December 2010–September 2022 (Figure 1). Search terms in PubMed and Google Scholar included coccidioidomycosis, cavitary, coccidioidal, cavitation, and cavity.

- Inclusion Criteria:
  - Age greater than 18 years old;
  - Radiographic evidence of cavitary lesion(s) with or without concurrent infiltrative disease;
  - Positive coccidioidal diagnosis based on one or more of the following:
    - Serology at the Kern County Public Health Department or the University of California Davis Mycology laboratories for immunodiffusion IgG and/or Compliment fixation (Immunodiffusion IgM alone or EIA results were not qualifying);
    - Positive respiratory secretion culture for *Coccidioides* spp.;
    - Positive histopathology demonstrating endosporulating spherules;
- Exclusion criteria:
  - Inadequate documentation for analysis;
  - Patients with pulmonary tuberculosis;
  - Incarcerated patients;
- Demographics and comorbidities:
  - Age at diagnosis;
  - Sex;
  - Race/Ethnicity;
  - Smoking tobacco;
  - Immunosuppressive medications;
  - HIV;
  - Diabetes Mellitus;
  - Chronic Obstructive Pulmonary Disease;
- Parameters that were used to investigate this study:
  - Simple cavity and location *;
  - Multiple cavities and locations *;
  - Cavity rupture (empyema) and associated intervention **;
  - Antifungal therapy;
  - Surgical intervention for hemorrhage and non-hemorrhagic etiologies;
  - Hemorrhage and associated intervention(s) (medical, surgical, interventional radiology).

* The concept of fibrocavity disease is not included due to difficulty with its definition. ** The definition of coccidioidal empyema is hydropneumothorax that results from cavitary rupture. Coccidioidal effusion is a part of primary coccidioidomycosis and, in rare instances, a manifestation of disseminated disease. Coccidioidal effusion is an entirely different entity and rarely requires a chest tube or surgical intervention [17].

## 3. Results

**Figure 2:**
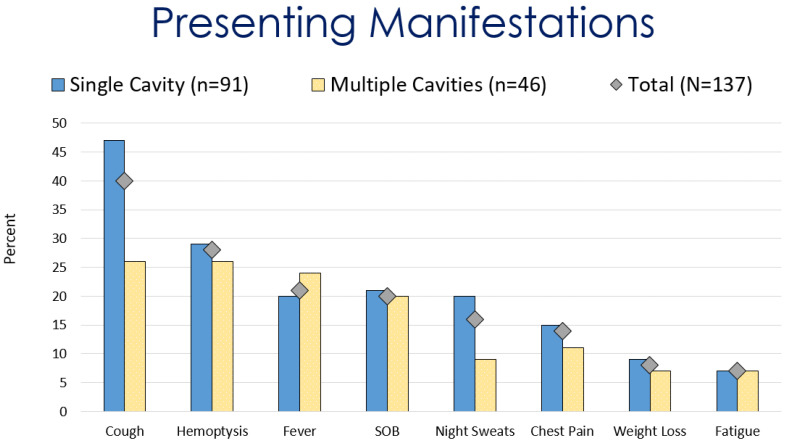
Comparison of presenting manifestations for patients with coccidioidal pulmonary cavitations.

Of the initially 276 identified patients, 137 met the inclusion criteria. At diagnosis, the most common presenting symptoms of pulmonary coccidioidal cavitary disease were cough (55%), hemoptysis (38%), fever (29%), and shortness of breath (28%) (Figure 2. Other symptoms included chest pain (19%), weight loss, and decreased appetite (11%). Twenty-one cases (15.3%) were immunocompromised/transplant patients. Twenty-six (19%) cases of cavitary lung disease were coexistent with disseminated coccidioidomycosis (Table 1).

**Figure 3:**
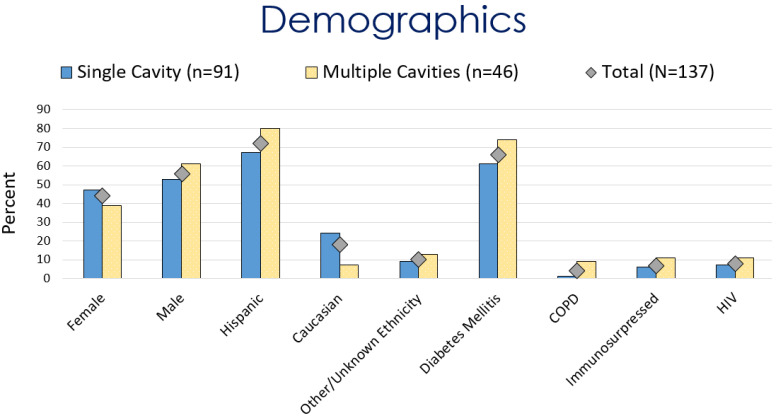
Comparison of demographics for patients with coccidioidal pulmonary cavitations.

**Figure 4:**
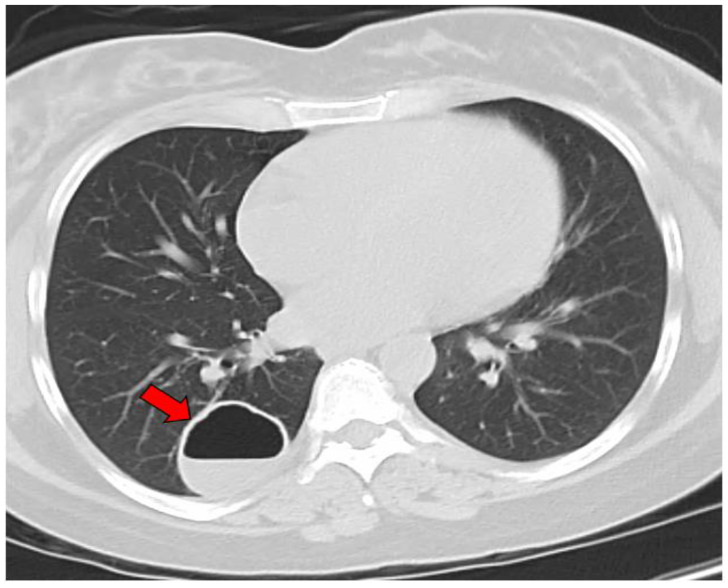
CT image of a single coccidioidal pulmonary cavity. The red arrow indicates a cavity.

**Figure 5:**
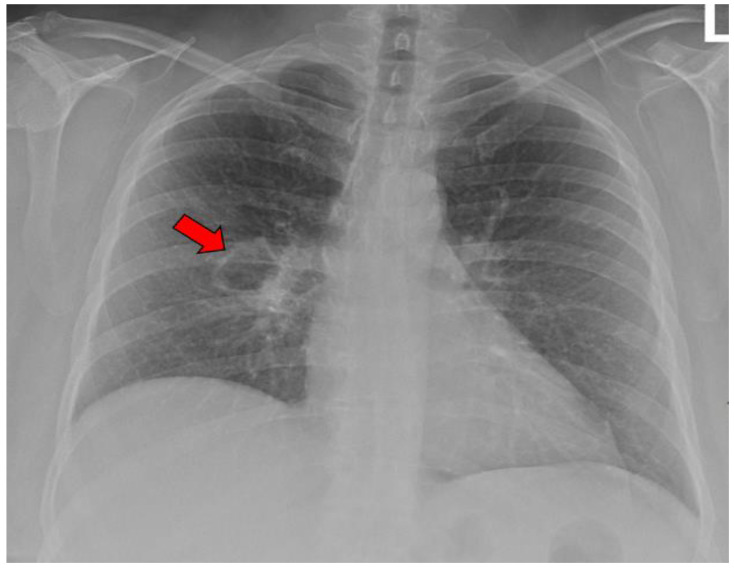
X-ray image of a single coccidioidal pulmonary cavity. The red arrow indicates a cavity.

**Figure 6:**
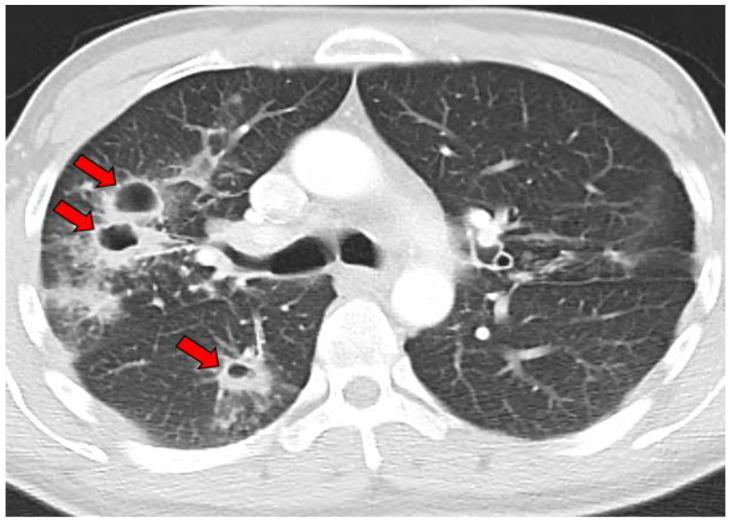
CT image of multiple coccidioidal pulmonary cavities. The red arrows indicate cavities.

**Figure 7:**
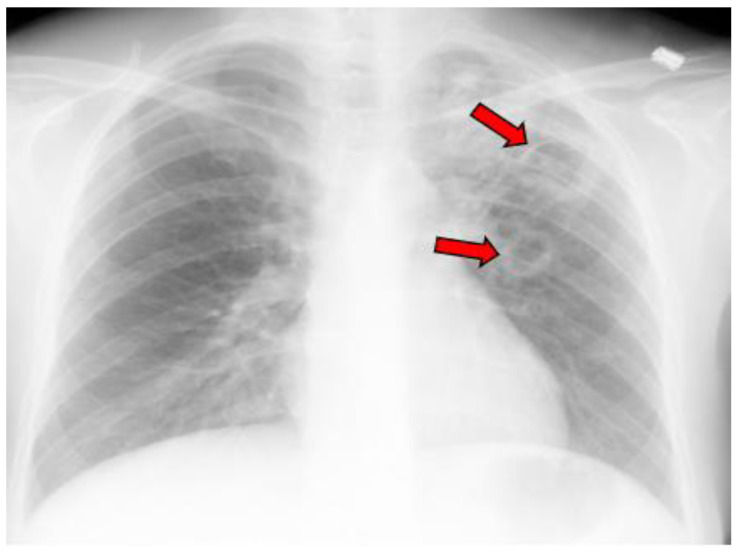
X-ray image of multiple coccidioidal pulmonary cavities. The red arrows indicate cavities.

The average age at the diagnosis of a cavitary lesion was 43.1 years old, ranging from 18 to 77 years old. There were more male patients (55.5%) than female patients (44.5%) (Figure 3). The most common radiographic finding was the presence of a single cavity (91/137, 66.4%). Examples of single cavities are provided in Figure 4 and Figure 5. Multiple cavities made up 33.6% of cases. Examples of cases with multiple cavities can be seen in Figure 6 and Figure 7. The most common locations for cavities were the upper lobes of the lungs (91/137, 66.4%) (Table 1).

The initial medical treatment for the majority of patients (122/137, 89%) was triazoles for a variable duration (Table 2). Fluconazole was used as the initial triazole in all but one patient with dosages ranging from 400–1000 mg. Five patients (3.7%) initially received an amphotericin B formulation, either for pulmonary or disseminated disease. Three patients (2.2%) received no medical therapy. Of these three patients, one was pregnant, another chose no treatment, and one died of complications due to cirrhosis before receiving treatment.

A total of nine patients were reported as deceased. The cause of death was either unknown or not directly related to pulmonary cavitation in all cases.

After the exclusion of disseminated coccidioidal cases and patients with insufficient data, the mean duration of the initial antifungal treatment was 563 days (n = 80). In 35% (28/80) of cases a triazole was switched to another triazole for variable reasons, including treatment failure or side effects. The switch from one antifungal treatment to another was considered the end of the initial treatment.

This study found 52 (37.2%) patients with hemoptysis. One case (0.7%) required radiologic intervention to occlude the bleeding vessel, and one (0.7%) case of hemorrhage required right upper-lobe lobectomy. No patients in this study required a Fogarty catheter.

Nine (6.6%) cases exhibited a ruptured cavity. Eight of those cases had initial chest tube placement, of which three (3/8, 37.5%) did not require surgical intervention. The remaining five (5/8, 62.5%) cases with an initial chest tube placement ultimately led to a thoracotomy or VATS (three (3/8, 37.5%) wedge resections, four (4/8, 50%) decortications, one (1/8, 12.5%) pleurodesis, and one (1/8, 12.5%) pneumonectomy).

Seven of 137 (5.1%) cases presented with a pleural effusion not associated with a cavity rupture. Five (3.7%) were due to primary coccidioidomycosis. The remaining two (1.5%) cases were attributed to heart failure. One of these two cases had a concurrent perinephric abscess with an associated exudative pulmonary effusion. This was the only cause of a non-coccidioidal exudative effusion in this study. Three of the coccidioidal effusions required therapeutic thoracentesis, and none required a chest tube or surgery.

## 4. Discussion

Recent research on cavitary coccidioidomycosis has focused on cavitary management in a largely Caucasian demographic and has shown an association with diabetes mellitus type 2 (DM) [15,16]. Recent data published by Crum et al. reviewed 223 patients from 1994–2002, of which 29 (13%) of coccidioidomycosis cases had coccidioidal pulmonary cavities, with only one requiring surgery for empyema [18]. In 2006, Santelli et al. concluded that patients with DM were more likely to have cavitary lung disease after reviewing 329 patients with coccidioidomycosis [15]. Later, Panicker et al. focused on the influence of triazole therapy on cavitary closure in 271 cavitary patients from a single site in Arizona [16].

Apart from the study of Panicker et al., this is the largest study of cavitary lung disease secondary to coccidioidomycosis in more recent times [16]. In some significant respects, however, our study differs from Panicker et al.’s and other historical research. By analyzing data from a large population of ethnically Hispanic patients, we studied a distinct demographic compared to those formerly reviewed by Panicker et al. and others. In addition, we also reviewed far fewer immunosuppressed patients or transplant recipients compared to the 106 (39.1%) in the Panicker et al. study [16].

A historical literature review in this field suggests that surgical techniques once used to manage cavitary tuberculosis, antecedent to antitubercular medical therapy, were also employed to treat coccidioidal disease. Conversely, this study and other recent research demonstrate the diminishing role of surgery in the management of coccidioidal cavities [16,18]. Compared to prior historical studies, such as that of Hyde, the care of these patients has evolved from aggressive surgical intervention to predominantly medical therapies [9]. This is further illustrated by our series’ considerably lower surgical rate when compared to Panicker’s (42 of 313, 13.4%) and much lower than the historical series from Hyde [9,16].

The Infectious Disease Society of America’s guideline recommends oral triazoles for symptomatic chronic cavitary coccidioidal pneumonia for at least one year; however, 30% have a recurrence of symptoms when treatment is discontinued [10]. This study’s mean duration of 563 days of initial treatment with a triazole suggests a longer duration of treatment may be indicated. The 35% of cases that switched initial triazole therapy to a different triazole suggest the need for close follow-up and therapeutic drug monitoring to distinguish between treatment failure and medication non-adherence.

This study found that some patients with pulmonary coccidioidal cavity complications were treated with less invasive interventions. If a ruptured cavity is diagnosed early, a chest tube may suffice in expanding the lung and sealing the leak. The chest tube may evacuate the air from the pleural space and restore negative intrathoracic pressure, which, in turn, may expand the lung and reestablish physiologic ventilation [19]. This is similar to the treatment of bacterial empyema described by Redden et al. in their meta-analysis [20]. If the ruptured cavity diagnosis is delayed, decortication, wedge resection, lobectomy, or even pneumonectomy may be needed. Tube thoracostomy and surgery are not mutually exclusive. In some cases, while tube thoracostomy may resolve the pneumothorax, a persistent air leakage requires surgical intervention.

Surgery may be required for life-threatening hemorrhage or the management of a cavity rupture into the pleural space with VATS or thoracotomy. However, this series demonstrates that surgery is utilized much less commonly when compared to historical cohorts. We hypothesize three reasons this may have occurred. First, the number of specialists has increased; thus, who evaluates, treats, and directs patient care has changed. Second, the advent of triazoles has provided a much more effective alternative medical treatment [16]. Lastly, the philosophies of thoracic surgeons have shifted from aggressive surgery to lung preservation [21].

The findings of this study indicate that pleural fluid in a patient with pulmonary cavitary coccidioidomycosis can be attributed to the following three reasons: cavity rupture (Section IV in Methods), coccidioidal effusion, or a non-coccidioidal disease process.

This study’s review contradicts the notion that pulmonary coccidioidal cavitary disease and dissemination infrequently manifest in the same patient (Table 2).

The inclusion and exclusion criteria for this study were stringent. Therefore, we inevitably excluded some patients with coccidioidal cavitary disease. This would include individuals with negative serology, negative histopathology, and negative culture that did not meet the inclusion criteria but had coccidioidal cavitary lung disease. It is also possible that people with a similar cavitary disease could have been falsely diagnosed as coccidioidal based on false positive serology.

Coccidioidal pulmonary cavitation remains a complex disease to evaluate and treat. In the present age of triazole therapy, indications and the need for surgery continue to decline. Further investigation needs to be conducted to evaluate medical therapy’s efficacy and long-term outcomes.

## Figures and Tables

**Table 1:** Demographic characteristics of 137 patients with cavitary coccidioidal lesions.

Characteristic		Single Cavity (n = 91)	Multiple Cavities (n = 46)	Total (n = 137)
Demographics and Comorbidities				
	Female	43 (47.3%)	18 (39.1%)	61 (44.5%)
	Hispanic	61 (67%)	37 (80.4%)	64 (46.7%)
	Caucasian	22 (24.2%)	3 (6.5%)	25 (18.2%)
	Black	3 (3.3%)	2 (4.3%)	5 (3.6%)
	Filipino	1 (1.1%)	1 (2.2%)	2 (1.5%)
	Middle Eastern	1 (1.1%)	0	1 (0.7%)
	Unknown Ethnicity	3 (3.3%)	3 (6.5%)	6 (4.4%)
	Diabetes Mellitus	56 (61.5%)	34 (73.9%)	90 (65.7%)
	Smoking Tobacco	21 (23.1%)	6 (13%)	27 (19.7%)
	Documented COPD *	1 (1.1%)	4 (8.7%)	5 (3.6%)
	Immunosuppressive medications	5 (5.5%)	5 (10.9%)	10 (7.3%)
	Transplant	0	0	0
	HIV	6 (6.6%)	5 (10.9%)	11 (8.0%)
Presenting Manifestations				
	Cough	43 (47.3%)	12 (26%)	55 (40.1%)
	Hemoptysis	26 (28.6%)	12 (26%)	38 (27.7%)
	Fever	18 (19.8%)	11 (23.9%)	29 (21.2%)
	Shortness of Breath	19 (20.9%)	9 (20%)	28 (20.4%)
	Night Sweats	18 (19.8%)	4 (8.7%)	22 (16.1%)
	Chest Pain	14 (15.4%)	5 (10.9%)	19 (13.9%)
	Weight Loss/Decreased Appetite	8 (8.8%)	3 (6.5%)	11 (8%)
	Fatigue	6 (6.6%)	3 (6.5%)	9 (6.6%)
Location **				
	Right Upper Lobe	31 (34.1%)	16 (34.8%)	47 (32.8%)
	Left Upper Lobe	26 (28.6%)	18 (39.1%)	44 (32.1%)
	Right Lower Lobe	18 (19.8%)	5 (10.9%)	23 (16.8%)
	Left Lower Lobe	13 (14.3%)	4 (8.7%)	17 (12.4%)
	Right Middle Lobe	3 (3.3%)	3 (6.5%)	6 (4.4%)

* Chronic Obstructive Pulmonary Disease. ** Multiple Cavities Location refers to the largest cavity.

**Table 2:** Concomitant dissemination sites if present, medical treatment, and complications among 137 patients with pulmonary coccidioidal cavitary lesions.

Characteristic		Single Cavity (n = 91)	Multiple Cavities (n = 46)	Total (n = 137)
Dissemination				
	Osseous	9 (9.9%)	2 (4.3%)	11 (8%)
	CNS	6 (6.6%)	1 (2.2%)	7 (5.1%)
	Integumentary	3 (3.3%)	0	3 (2.2%)
	Other	5 (5.5%)	2 (4.3%)	7 (5.1%)
	Total	23 (25.3%)	5 (10.9%)	28 (20.4%)
Treatment				
	Fluconazole *	81 (89%)	41 (84.8%)	122 (89.1%)
	Other Triazole *	1 (1.1%)	0	1 (0.7%)
	Amphotericin *	4 (4.4%)	1 (2.2%)	5 (3.6%)
	None *	1 (1.1%)	2 (4.3%)	3 (2.2%)
	Unknown *	4 (4.4%)	2 (4.3%)	6 (4.3%)
	Surgery	4 (4.4%)	5 (10.9%)	9 (6.6%)
Complications				
	Hemoptysis	33 (36.3%)	18 (39.1%)	51 (37.2%)
	Superinfection	7 (7.7%)	5 (10.9%)	12 (8.8%)
	Pleural Effusion	1 (1.1%)	6 (13%)	7 (5.1%)
	Rupture, Empyema, or fistula	4 (4.4%)	5 (10.9%)	9 (6.6%)
	Pneumothorax	6 (6.6%)	4 (8.7%)	10 (7.3%)
	Fungal Ball **	3 (3.3%)	4 (8.7%)	7 (5.1%)

* Initial medical treatment. ** Mass within cavity presumed to be a fungal ball.

## Data Availability

Data supporting the results can be obtained by reaching the corresponding author.
